# Harder, better, faster, stronger? Retrospective chart review of adverse events of interactions between adaptogens and antidepressant drugs

**DOI:** 10.3389/fphar.2023.1271776

**Published:** 2023-09-27

**Authors:** Marcin Siwek, Jarosław Woroń, Anna Wrzosek, Jarosław Gupało, Adrian Andrzej Chrobak

**Affiliations:** ^1^ Department of Affective Disorders, Chair of Psychiatry, Jagiellonian University Medical College, Kraków, Poland; ^2^ Department of Clinical Pharmacology, Chair of Pharmacology, Faculty of Medicine, Jagiellonian University Medical College, Kraków, Poland; ^3^ Department of Anesthesiology and Intensive Care, University Hospital in Cracow, Kraków, Poland; ^4^ University Center for Monitoring and Research on Adverse Drug Effects in Krakow, Kraków, Poland; ^5^ Department of Interdisciplinary Intensive Care, Jagiellonian University, Krakow, Poland; ^6^ Pharma Consult, Pharmacotherapy Safety Team, Zakopane, Poland; ^7^ Department of Adult Psychiatry, Chair of Psychiatry, Jagiellonian University Medical College, Kraków, Poland

**Keywords:** ashwagandha, maca, jiaogulan, berberine, herb–drug interactions, depression, cytochrome, p-glycoprotein

## Abstract

**Aim:** We aimed to systematically evaluate the prevalence and clinical characteristics of adverse events associated with the adaptogens and antidepressant drug interactions in a retrospective chart review.

**Methodology:** A total of 1,816 reports of adverse events were evaluated. Cases were included in the analysis if the pharmacoepidemiological analysis showed the presence of a high probability of a causal relationship between an adaptogen and antidepressant interaction and the occurrence of adverse events. The following data were extracted from the reports: age, sex, antidepressant, plant products containing adaptogens, other concomitant medications, and clinical consequences of the interactions and their possible mechanisms.

**Results:** Adaptogens were involved in 9% of adverse events associated with the concomitant use of antidepressants and other preparations. We identified 30 reports in which side effects presented a causal relationship with the use of antidepressants and adaptogens. Here, we present the list of adaptogens with the corresponding antidepressants and the side effects caused by their interactions: *Withania somnifera*: reboxetine (testicle pain and ejaculatory dysfunctions), sertraline (severe diarrhea), escitalopram (myalgia, epigastric pain, nausea, vomiting, restless legs syndrome, and severe cough), and paroxetine (generalized myalgia, ophthalmalgia, and ocular hypertension); *Eleutherococcus senticosus*: duloxetine (upper gastrointestinal bleeding), paroxetine (epistaxis), sertraline (vaginal hemorrhage), and agomelatine (irritability, agitation, headache, and dizziness); *Schisandra chinensis*: bupropion (arthralgia and thrombocytopenia), amitriptyline (delirium), and fluoxetine (dysuria); *Tribulus terrestris*: citalopram (generalized pruritus), escitalopram (galactorrhea), and trazodone (psoriasis relapse); *Coptis chinensis*: mianserin (arrhythmias), mirtazapine (edema of lower limbs and myalgia), and fluoxetine (gynecomastia); *Cimicifuga racemosa*: mianserin (restless legs syndrome), paroxetine (gynecomastia and mastalgia), and venlafaxine (hyponatremia); *Bacopa monnieri*: agomelatine (back pain and hyperhidrosis) and moclobemide (myocardial infarction); *Gynostemma pentaphyllum*: duloxetine (back pain); *Cordyceps sinensis*: sertraline (upper gastrointestinal bleeding); *Lepidium meyenii*: mianserin (restless legs syndrome); and *Scutellaria baicalensis*: bupropion (seizures).

**Conclusion:** Clinicians should monitor the adverse events associated with the concomitant use of adaptogens and antidepressant drugs in patients with mental disorders. Aggregation of side effects and pharmacokinetic interactions (inhibition of CYP and p-glycoprotein) between those medicines may result in clinically significant adverse events.

## 1 Introduction

Adaptogens are defined as non-toxic substances of plant origin that are claimed to increase “non-specific” resistance to a broad spectrum of adverse biological, chemical, and physical factors, normalize body functions, and strengthen the system compromised by stress (Committee on Herbal Medicinal Products, 2008). The broad and vague definition of the term renders it of little scientific value. It is difficult to determine the minimum requirements needed for “strengthening” such a preparation. Therefore, almost every plant preparation, with which some positive effects have been indicated, can be called an adaptogen. As the principle of an adaptogenic action needs further clarification, this term is not accepted in clinical and pharmacological terminologies in the European Union and has been considered not appropriate for marketing authorization (Committee on Herbal Medicinal Products, 2008). Nevertheless, in this article, we have decided to use the term “adaptogen,” as we believe it would make it easier for physicians and patients to find the results of our study. In the literature, more than 100 plants have been described as having “adaptogenic properties” (Panossian et al., 2021), of which the most extensively studied are *Withania somnifera* (ashwagandha), *Schisandra chinensis*, *Rhodiola rosea*, and *Eleutherococcus senticosus* (Panossian, 2013; Todorova et al., 2021). As the adaptogens are obtainable without prescriptions, their use has become increasingly popular. For example, according to the National Institutes of Health Office of Dietary Supplements, there are currently more than 1,300 products containing *Withania somnifera* in the United States markets alone (Speers et al., 2021), and by 2019, preparations derived from this plant had become the fifth most popular dietary supplement (Smith et al., 2020). An increasing number of studies suggest that adaptogens may alleviate fatigue, insomnia, anxiety, memory impairments, and depressive symptoms and reduce the level of perceived stress (Panossian and Wikman, 2009a; Panossian, 2013; Panossian et al., 2021; Todorova et al., 2021). Thus, these substances are commonly used by patients suffering from mental disorders, who take them along with their medication, as a form of complementary treatment or with the aim to ameliorate side effects experienced during psychopharmacotherapy. Although adaptogens are non-toxic and generally well tolerated, they still may induce adverse interactions with other drugs. Notably, plant preparations usually consist of numerous, separate, pharmacologically active substances that function as independent drugs. For instance, more than 40 withanolides, approximately 12 alkaloids, and several sitoindosides have been isolated from *Withania somnifera* (Mirjalili et al., 2009). Such a large group of bioactive compounds may significantly increase the risk of adverse events (Woroń and Siwek, 2018).

Patients treated for mental disorders are already exposed to the side effects associated with the use of polytherapy, which is defined as the use of at least two drugs at the same time. Approximately one-third of the patients in the United States are treated with at least three psychotropic medications, and this proportion has been shown to be increasing over time (Mojtabai and Olfson, 2010). Even the use of two drugs at the same time poses the risk of adverse interactions, and if seven drugs are taken simultaneously, the occurrence of such interactions is certain (Vickers et al., 2006; McIntyre et al., 2016; Schatzberg and Nemeroff, 2017; Woroń and Siwek, 2018). One of the most frequently used psychotropic drugs are antidepressants (Brody and Gu, 2015). Apart from major depressive disorder, these medicines are used to treat anxiety disorders, insomnia, eating disorders, or chronic pain. As adaptogens are suggested to alleviate the symptoms that occur in those conditions, the concomitant use of those preparations and antidepressants may be a common phenomenon. Despite the high popularity of adaptogens and the frequent use of antidepressants, adverse interactions between those two groups of substances have not been extensively studied. While the use of adaptogens in combination with other drugs is considered to be low risk, the data supporting those claims come from animal/*in vitro* studies and from the research conducted on a small group of patients that did not implement methodology specifically addressing this issue (Tandon and Yadav, 2020; Fuladi et al., 2021). As the adaptogens are registered as dietary supplements, their interactions with other drugs are not rigorously monitored by the United States Food and Drug Administration. Thus, there is an urgent need to systematically evaluate the risks associated with the use of those preparations during psychopharmacotherapy.

The aim of this research is to systematically evaluate the characteristics and incidence of adverse events associated with the concomitant use of adaptogens and antidepressant drugs in a retrospective chart review.

## 2 Materials and methods

In order to evaluate the prevalence and clinical characteristics of adverse events associated with the concomitant use of adaptogens and antidepressants, we performed a retrospective chart review according to the methodology of our previous studies on psychotropic drug interactions (Woroń and Siwek, 2018; Woroń et al., 2019; Siwek et al., 2020). All authors performed the analysis. The dataset consisted of reports on the occurrence of adverse reactions caused by the interactions between simultaneously used drugs. The reports were analyzed at the University Center for Monitoring and Research on Adverse Drug Effects, Department of Clinical Pharmacology, Jagiellonian University Medical College, Cracow. This unit has been authorized by Polish legal acts to formally monitor and report adverse events related to pharmacotherapy, as well as to provide pharmacological consultations for clinics and hospitals in the Silesian, Subcarpathian, Lesser Poland, and Holy Cross regions. Due to the increasing number of reported side events associated with the use of psychotropic drugs, this unit cooperates with the Department of Affective Disorders of Jagiellonian University Medical College. Approximately 850–1,100 consultations are made per year (Woroń et al., 2022).

In the current study, we have evaluated reports that were received from all over Poland in the period between January 2021 and November 2022. The analyzed period was selected on the basis of the availability of the data. The first reports of side effects related to the use of adaptogens were found in January 2021. The cases were included in the study when the following criteria were met: 1) patients used at least one antidepressant drug, 2) patients received at least one adaptogen, and 3) the presence of a high probability of a causal relationship in terms of pharmacodynamic interactions, pharmacokinetic interactions, or the interactions associated with the aggregation of side effects caused by the concomitant use of adaptogens and antidepressant drugs indicated by the pharmacoepidemiological analysis. The cause-and-effect relationship was indicated when the following two conditions were met: 1) the mechanism of interactions leading to the described adverse events may be demonstrated on the basis of the existing literature; 2) the discontinuation of products containing adaptogenic plant extracts resulted in the amelioration of the described side effects.

**FIGURE 1:**
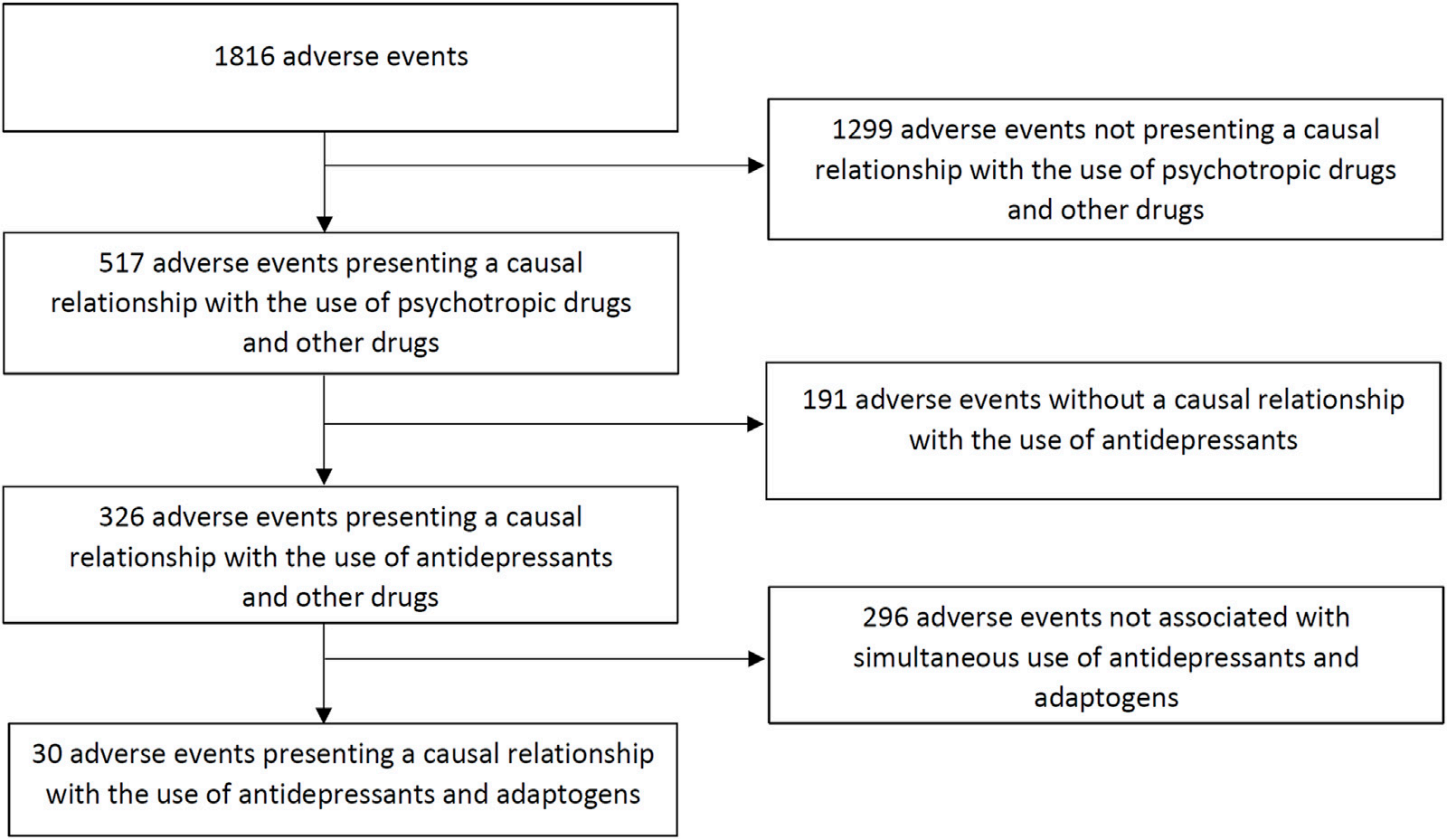
Flow chart of the retrospective chart review.

Figure 1 shows a flow chart of our retrospective chart review. We evaluated 1,816 registered adverse events, of which 517 presented a causal relationship with the use of psychotropic medication. A total of 326 adverse events were associated with the use of antidepressants, of which 30 were caused by the simultaneous use of medical products containing adaptogens (9%).

## 3 Results

Table 1 summarizes the data extracted from 30 adverse events causally related to the simultaneous use of adaptogens and antidepressant drugs. The mean age of the patients described in the reports was 57 ± 14.3 years. The reports included 17 women and 13 men. The group of antidepressants that showed the highest rates of adverse events was serotonin reuptake inhibitors (SSRIs, 14 patients, 46%), which involved escitalopram (five patients, 17%), sertraline (three patients, 10%), paroxetine (three patients, 10%), fluoxetine (two patients, 7%), and citalopram (one patient, 3%). Three cases (10%) demonstrated adverse events associated with the use of serotonin and norepinephrine reuptake inhibitors (SNRIs), particularly two patients (7%) treated with duloxetine and one patient (3%) with venlafaxine. Other antidepressants presenting adverse interactions with adaptogens were as follows: reboxetine (one patient, 3%), bupropion (three patients, 10%), trazodone (one patient, 3%), mianserin (three patients, 10%), mirtazapine (one patient, 3%), amitriptyline (one patient, 3%), agomelatine (two patients, 7%), and moclobemide (one patient, 3%). In the case of adaptogens, interactions involved *Withania somnifera* (seven patients, 23%), *Eleutherococcus senticosus* (four patients, 13%), *Schisandra chinensis* (four patients, 13%), *Tribulus terrestris* (three patients), *Coptis chinensis* (three patients, 10%), *Cimcifuga racemosa* (three patients, 10%), *Bacopa monnieri* (two patients, 7%), *Gynostema pentaphyllum* (one patient, 3%), *Cordyceps sinensis* (one patient, 3%), *Lepidium meyenii* (one patient, 3%), and *Scutellaria baicalensis* (one patient, 3%). Most of the analyzed adverse events resulted from pharmacokinetic interactions (20 reports, 67%). In the case of two patients (7%), they were caused by the addition of side effects. Eight adverse events were of mixed origin (27%, the presence of both pharmacokinetic interactions and the addition of side effects). A detailed description of the proposed mechanisms and their clinical consequences are shown in Table 1.

**TABLE 1:** Interactions between adaptogens and antidepressant drugs in the analyzed group and possible interaction mechanisms. p-gp, p-glycoprotein.

Plant products containing adaptogens	Antidepressant medication	Sex/age	Other concomitant medications	Clinical consequences of the interaction	Possible interaction mechanism
*Withania somnifera*	Reboxetine	M/56	Perindopril, amlodipine, and metformin	Testicle pain, ejaculatory dysfunctions, and pain during ejaculation	Pharmacokinetic: inhibition of CYP3A4 and CYP2D6 by *Withania somnifera* increased concentration and side effects of reboxetine (metabolized by CYP2D6 and CYP3A4)
	Sertraline	M/36	Doxylamine	Severe diarrhea requiring hospitalization	Addition of side effects: *Withania somnifera* may induce gastrointestinal symptoms, including diarrhea; 20% of patients treated with sertraline present diarrhea
					Pharmacokinetic: Inhibition of p-gp by *Withania somnifera* increased concentration and side effects of sertraline which is a p-gp substrate
	Escitalopram	M/58	Dapagliflozin, metformin, and zofenopril	Myalgia NRS>5	Pharmacokinetic: Inhibition of p-gp, CYP3A4, and CYP2D6 by *Withania somnifera* increased concentration and side effects of escitalopram (transported by p-gp and metabolized by CYP2D6 and CYP3A4)
	Escitalopram	F/64	Atorvastatin, metformin, perindopril, and indapamide	Epigastric pain, nausea, and vomiting	Addition of side effects: *Withania somnifera* may induce gastrointestinal symptoms including epigastric pain and nausea. The aforementioned symptoms are common side effects observed during escitalopram therapy
					Pharmacokinetic: Inhibition of p-gp, CYP3A4, and CYP2D6 by *Withania somnifera* increased concentration and side effects of escitalopram (transported by p-gp and metabolized by CYP2D6 and CYP3A4)
	Escitalopram	F/71	Formoterol, fluticasone, zofenopril, and hydrochlorothiazide	Restless legs syndrome	Pharmacokinetic: Inhibition of p-gp, CYP3A4, and CYP2D6 by *Withania somnifera* increased concentration and side effects of escitalopram (transported by p-gp and metabolized by CYP2D6 and CYP3A4)
	Escitalopram	F/58	Sitagliptin, metformin, atorvastatin, ezetimibe, and trazodone (50 mg/day)	Severe non-productive cough resistant to antitussive medication	Pharmacokinetic: Inhibition of p-gp, CYP3A4, and CYP2D6 by *Withania somnifera* increased concentration and side effects of escitalopram (transported by p-gp and metabolized by CYP2D6 and CYP3A4)
					Addition of side effects: *Withania somnifera* may induce cough which is one of the side effects observed during escitalopram therapy
	Paroxetine	F/31	Lorazepam	Generalized myalgia (NRS = 4–5), ophthalmalgia, and ocular hypertension	Pharmacokinetic: Inhibition of p-gp, CYP3A4, and CYP2D6 by *Withania somnifera* increased concentration and side effects of paroxetine (transported by p-gp and metabolized by CYP2D6 and CYP3A4)
*Eleutherococcus senticosus*	Duloxetine	F/68	Bisoprolol, aspirin, clopidogrel, zofenopril, and tamsulosin	Upper gastrointestinal bleeding	Addition of side effects: *Eleutherococcus senticosus*, due to its significant antiplatelet activity, may increase the risk of bleeding in the case of concomitant use of anticoagulant and antiplatelet drugs as well as SNRI
	Paroxetine	M/64	Ticagrelor, aspirin, perindopril, allopurinol, atorvastatin, and pantoprazole	Epistaxis	Addition of side effects: *Eleutherococcus senticosus*, due to its significant antiplatelet activity, may increase the risk of bleeding in the case of the concomitant use of anticoagulant and antiplatelet drugs as well as SSRI
					Pharmacokinetic: Inhibition of p-gp by *Eleutherococcus senticosus* increased the concentration and side effects of paroxetine which is a p-gp substrate
	Sertraline	F/41	Bisoprolol, rivaroxaban, propafenone, and pantoprazole	Vaginal hemorrhage	Addition of side effects: *Eleutherococcus senticosus*, due to its significant antiplatelet activity, may increase the risk of bleeding in the case of the concomitant use of anticoagulant and antiplatelet drugs as well as SSRI
					Pharmacokinetic: Inhibition of p-gp by *Eleutherococcus senticosus* increased the concentration and side effects of sertraline which is a p-gp substrate
	Agomelatine	F/39	Alprazolam and ethinyloestradiol + dienogest	Irritability, agitation, headache, and dizziness	Pharmacokinetic: *Eleutherococcus senticosus* inhibits p-gp, CYP2C9, and CYP1A2, increasing the concentration and side effects of agomelatine (transported by p-gp and metabolized by CYP2C9 and CYP1A2)
					Addition of side effects: Irritability and headache have been listed as some of the side effects observed during the use of *Eleutherococcus senticosus* as well as agomelatine
*Tribulus terrestris*	Citalopram	M/29	Lorazepam	Generalized pruritus	Pharmacokinetic: *Tribulus terrestris* inhibits CYP3A4, increasing the concentration and side effects of citalopram (metabolized by CYP3A4)
	Escitalopram	F/31	Zolpidem	Galactorrhea	Pharmacokinetic: *Tribulus terrestris* inhibits CYP3A4, increasing the concentration and side effects of escitalopram (metabolized by CYP3A4)
	Trazodone	M/39	Ramipril, indapamide, and metoprolol	Psoriasis relapse	Pharmacokinetic: *Tribulus terrestris* inhibits CYP3A4, increasing the concentration and side effects of trazodone (metabolized by CYP3A4). By this interaction, *Tribulus terrestris* may increase the risk of psoriasis relapse. This is of clinical significance if trazodone is used in daily doses above 300 mg when it reveals significant SSRI activity
*Schisandra chinensis*	Bupropion	M/42	Alprazolam	Generalized arthralgia	Pharmacokinetic: *Schisandra chinensis* inhibits CYP2B6, CYP2C9, CYP3A4, and CYP2E1, increasing the concentration and side effects of bupropion (metabolized by CYP2B6, CYP2C9, CYP3A4, and CYP2E1)
	Bupropion	M/40	Hydroxyzine	Thrombocytopenia	Pharmacokinetic: *Schisandra chinensis* inhibits CYP2B6, CYP2C9, CYP3A4, and CYP2E1, increasing the concentration and side effects of bupropion (metabolized by CYP2B6, CYP2C9, CYP3A4, and CYP2E1)
	Amitriptyline	F/79	Metoprolol, ramipril, torsemide, potassium, etoricoxib, and glucosamine sulfate	Delirium	Pharmacokinetic: *Schisandra chinensis* inhibits CYP3A4, CYP2C19, and CYP2C9, increasing the concentration and side effects of amitriptyline (metabolized by CYP3A4, CYP2C19, and CYP2C9)
	Fluoxetine	F/74	Perindopril, amlodipine, furosemide, and chondroitin sulfate	Dysuria	Pharmacokinetic: *Schisandra chinensis* inhibits CYP2C9, increasing the concentration and side effects of fluoxetine (metabolized by CYP2C9)
*Gynostema pentaphyllum*	Duloxetine	M/44	Melatonin	Lower back pain (NRS = 5–6) with increased muscular tension	Pharmacokinetic: *Gynostema pentaphyllum* inhibits CYP2D6, increasing the concentration and side effects of duloxetine (metabolized by CYP2D6)
*Cordyceps sinensis*	Sertraline	M/58	Ticagrelor, aspirin, ramipril, pitavastatin, bisoprolol, and dexlansoprazole	Upper gastrointestinal bleeding	Addition of side effects: *Cordyceps sinensis*, due to its significant antiplatelet activity, may increase the risk of bleeding in the case of concomitant use of anticoagulant and antiplatelet drugs as well as SSRIs
*Cimicifuga racemosa*	Mianserin	F/56	Bisoprolol	Restless legs syndrome	Pharmacokinetic: *Cimicifuga racemosa* inhibits CYP2D6 and CYP3A4, increasing the concentration and side effects of mianserin (metabolized by CYP2D6 and CYP3A4)
	Paroxetine	F/61	Lornoxicam and glucosamine sulfate	Gynecomastia and mastalgia (NRS>5)	Pharmacokinetic: *C. racemosa* inhibits CYP2D6, increasing the concentration and side effects of paroxetine (metabolized by CYP2D6)
					Addition of side effects: Breast pain/enlargement has also been observed during the use of *Cimcifuga racemosa* extracts
	Venlafaxine	F/58	Alprazolam, zofenopril, and lercanidipine	Hyponatremia	Pharmacokinetic: *Cimicifuga racemosa* inhibits CYP2D6 and CYP3A4, increasing the concentration and side effects of venlafaxine (metabolized by CYP2D6 and CYP3A4)
*Coptis chinensis*	Mianserin	M/60	Tamsulosin, budesonide, fluticasone, and theophylline	Ventricular arrhythmias	Pharmacokinetic: *Coptis chinensis* inhibits CYP2D6 and CYP3A4à increasing concentration and side effects of mianserin (metabolized by CYP2D6 and CYP3A4)
	Mirtazapine	F/48	Oxazepam and bilastine	Edema of lower limbs and myalgia	Pharmacokinetic: *Coptis chinensis* inhibits CYP3A4, increasing the concentration and side effects of mirtazapine (metabolized by CYP3A4)
	Fluoxetine	F/32	Alprazolam	Gynecomastia	Pharmacokinetic: *Coptis chinensis* inhibits CYP3A4, increasing the concentration and side effects of fluoxetine (metabolized by CYP3A4)
*Lepidium meyenii*	Mianserin	M/64	Doxazosin, perindopril, and indapamide	Restless legs syndrome	Pharmacokinetic: *Lepidium meyenii* inhibits CYP3A4, increasing the concentration and side effects of mianserine (metabolized by CYP3A4)
*Scutellaria baicalensis*	Bupropion	F/67	Chondroitin sulfate, tramadol, sertraline, and etoricoxib	Seizures	Pharmacokinetic and addition of side effects: *Scutellaria baicalensis* strongly inhibits CYP2C9, increasing the concentration and side effects of bupropion (metabolized by CYP2C9). As tramadol, sertraline, and bupropion decrease the seizure threshold, increased concentration of the latter one leads to more severe outcomes of the addition of side effects
*Bacopa monnieri*	Agomelatine	F/42	Sitagliptin, metformin, and bisoprolol	Back pain and hyperhidrosis	Pharmacokinetic: *Bacopa monnieri* inhibits p-gp, CYP1A2, CYP2C9, and CYP2C19, increasing the concentration and side effects of agomelatine (transported by p-gp, and metabolized by CYP1A2, CYP2C9, and CYP2C19)
	Moclobemide	M/39	Doxylamine, metformin, dapagliflozin, and rosuvastatin	Myocardial infarction	Pharmacokinetic: *Bacopa monnieri* inhibits CYP2C19, increasing the concentration and side effects of moclobemide (metabolized by CYP2C19)

According to the reports, the discontinuation of products containing adaptogenic plant extracts led to the amelioration of the described symptoms. Corrective therapy was required for severe adverse reactions. In all the described cases, a causal relationship was established between the combination of the drug and the product containing plant extracts and the side effects that the patient experienced.

## 4 Discussion

To the best of our knowledge, this is the first retrospective chart review evaluating the prevalence and clinical characteristics of the adverse events associated with the concomitant use of adaptogens and antidepressant drugs. A thorough evaluation of 326 reports showed that 9% of adverse events caused by interactions of antidepressants with other drugs were most likely caused by their concomitant use with adaptogens, particularly *Withania somnifera*, *Eleutherococcus senticosus*, *Schisandra chinensis, Tribulus terrestris*, *Coptis chinensis*, *Cimcifuga racemosa*, *Bacopa monnieri*, *Gynostema pentaphyllum*, *Cordyceps sinensis*, *Lepidium meyenii*, and *Scutellaria baicalensis.* Notably, in all of the cases, discontinuation of the adaptogenic preparations led to remission of the described symptoms. Table 2 shows the side effects associated with the use of those adaptogens and their effects on cytochrome P450 and p-glycoprotein. Table 3 presents the relationships between antidepressants drugs and cytochrome isoenzymes and p-glycoprotein.

**TABLE 2:** Side effects and possible interaction mechanisms of the analyzed adaptogens. ↑ indicating induction and ↓ indicating inhibition.

		Side effect		Interactions with cytochrome	Interactions with p-glycoprotein
*Withania somnifera*		Somnolence, epigastric pain/discomfort, diarrhea, giddiness, drowsiness, hallucinations, vertigo, rhinitis, cough, cold, decreased appetite, nausea, constipation, dry mouth, hyperactivity, nocturnal cramps, blurring of vision, hyperacidity, skin rash, and weight gain (Tandon and Yadav, 2020)		↑ ↓/none CYP3A4	↓ (Hassan et al., 2021)
				↓/none CYP2D6	
				↓ CYP2B6	
				↑ CYP1A2	
				(Brinker (2010); Savai et al. (2013); Savai et al. (2015); Sultana and Sultan (2018); Kumar et al. (2021b); Haron et al. (2022))	
*Eleutherococcus senticosus*		Insomnia, shifts in heart rhythm, tachycardia, extrasystoles, palpitations, headache, pericardial pain, elevated blood pressure, irritability, melancholy, anxiety, and bleeding (European Medicine’s Agency, 2008)		↓ CYP2C9	↓ (Takahashi et al., 2010)
				↓ CYP2E1	
				(Guo et al., 2014)	
*Schisandra chinensis*		Dyspepsia, anorexia, urticaria, restlessness, insomnia, and dyspnea (Bove et al., 2010; St. John, 2018)		↓ CYP3A4	↓ (F. Zhang et al., 2022)
				↓ CYP2B6	
				↓ CYP2C8	
				↓ CYP2C9	
				↓ CYP2C19	
				↓ CYP2E1	
				(Jiang et al., 2010; Seo et al., 2021b)	
*Tribulus terrestris*		Abdominal pain/distension/discomfort, diarrhea, gastric upset, halitosis, headache, insomnia, irritability, nausea, and priapism (Gama et al., 2014; Campanelli et al., 2016)		↓ CYP3A4	—
				(Wang et al., 2022)	
*Coptis chinensis*		—		↓ CYP3A4	↑ (Yu et al., 2018)
				↓ CYP2C9	
				↓ CYP2D6	
				(Guo et al., 2012; Yu et al., 2018)	
*Cimcifuga racemosa*		Stiffening of extremities, gastric pain, allergic reactions, gastrointestinal symptoms (dyspeptic disorders and diarrhea), facial edema, and peripheral edema (EMA, 2017)		↓ CYP3A4,	Not significant (Gurley et al., 2006)
				↓ CYP2D6	
				(J. Li et al., 2011; Tsukamoto et al., 2005)	
*Bacopa monnieri*		Gastrointestinal symptoms (increased stool frequency, nausea, and abdominal cramps) (Walker and Pellegrini, 2023)		↓ CYP2C9	↓ (Singh et al., 2013)
				↓ CYP2C19	
				↓ CYP1A2	
				↓ CYP2D6	
				↓ CYP3A4	
				(Ramasamy et al., 2014)	
*Gynostema pentaphyllum*		Nausea and increased bowel movements (Chen and Su, 1989)		↓ CYP2D6	↓ (Zhu et al., 2012)
				↓ CYP2C8	
				↓ CYP3A4	
				↓ CYP2C9	
				(He et al., 2013)	
*Cordyceps sinensis*		Bleeding, dry mouth, abdominal distension, throat discomfort, and headache (Hatton et al., 2018; X. Yu et al., 2019)		—	—
*Lepidium meyenii*		—		↓/none CYP3A4	—
				(Brinker, 2010; Ibrahim et al., 2022; Y. Zhang et al., 2019)	
*Scutellaria baicalensis*		Skin allergy, platelet count reduction, pneumonia, and liver damage (Kiguchi et al., 2000; Takeshita et al., 2001; Dhanasekaran et al., 2013; Lin et al., 2016)		↓ CYP2C9	↓ (Miao et al., 2016)
				↑ CYP2E1	
				↓ CYP2C19	
				↓CYP4F2	
				↓/↑ CYP3A4	
				↓/↑ CYP2B6	
				↓ CYP1A2	
				(Zhou et al., 2021)	

**TABLE 3:** Antidepressants as substrates of cytochrome P450 (CYP450) and p-glycoprotein (p-gp). X–effects shown in animal and human studies; ((X))—effect shown in animal studies but is not confirmed in human and human cell studies; X!—strong effect; X?—effect demonstrated in animal studies, but no clinical trials or studies on human cells have been conducted so far (Siwek, 2023).

	CYP450 substrate	P-gp substrate
Agomelatine	1A2 > 2C19/2C9	X
Amitriptyline	2D6/3A4/2C19	((X))
	>1A2/2C9/2B6/2C8	
Bupropion	2B6 > 2E1	—
Citalopram	2C19 > 3A4/2D6	X
Duloxetine	1A2/2D6 >2C9	—
Escitalopram	2C19 >3A4/2D6	X
Fluoxetine	2D6 >3A4/3A5,	((X))
	/2C9/2C19, 2B6/1A2	
Mirtazapine	3A4 >2D6/1A2	
Moclobemide	2C19> 2D6	
Paroxetine	2D6 >3A4/1A2/2C19	X
Reboxetine	3A4 > 2D6	—
Sertraline	2C19 > 2C9, 3A4	X!
Trazodone	3A4 > 2D6, 1A2	X
Venlafaxine	2D6 > 3A4	—
Mianserin	2D6 > 3A4	—

*Withania somnifera* was associated with the highest number of adverse events caused by the simultaneous use of antidepressants and adaptogens, presumably because it is one of the most commonly used dietary supplements (Smith et al., 2020). Interactions were mainly associated with the use of SSRIs. Proposed mechanisms underlying those events involve: 1) interactions between adaptogen and cytochrome 450 isoenzymes responsible for antidepressant metabolism and 2) the addition of side effects of both substances. The first mechanism is related to the suggested inhibitory effect of *Withania somnifera* extracts on CYP3A4 and CYP2D6 (Brinker, 2010; Sultana and Sultan, 2018). This results in an increase in the concentration and side effects of antidepressants metabolized by those cytochromes, particularly escitalopram (myalgia of intensity >5 according to the numeric rating scale (NRS), epigastric pain, nausea, vomiting, restless legs syndrome, and severe non-productive cough), paroxetine (generalized myalgia (NRS = 4–5), ophthalmalgia, and ocular hypertension), and reboxetine (testicle pain, ejaculatory dysfunctions, and pain during ejaculation). The addition of the side effects was involved in the occurrence of severe diarrhea requiring hospitalization in a patient treated with sertraline and with the presence of epigastric pain, nausea, and vomiting in the case of an individual treated with escitalopram. Those two drugs are one of the least tolerated antidepressants in terms of gastrointestinal side effects (Oliva et al., 2021). For example, diarrhea was presented in up to 20% of patients receiving sertraline (Sertraline Side Effects, 2023). Loose stools and epigastric pain were also reported as one of the most common side effects (>5%) among individuals receiving *Withania somnifera* (Tandon and Yadav, 2020). Therefore, the overlap of those symptoms caused by the use of *Withania somnifera*, sertraline, and escitalopram are plausible explanations for observed adverse events. To the best of our knowledge, there are no studies on herb–drug interactions between this adaptogen and antidepressant drugs. One *in vitro* study suggests that *Withania somnifera* extracts have the potential to cause clinically significant herb–drug interactions through their associations with CYP3A4 and CYP2B6 metabolism pathways (Kumar et al., 2021a). However, there are conflicting results concerning the nature of those interactions. *In vitro* studies reported that *Withania somnifera* extracts may inhibit (Sultana and Sultan, 2018), induce (Kumar et al., 2021a), or reveal no significant impact on CYP3A4 (Savai et al., 2013, Savai et al., 2015). Further studies are required to understand the associations between CYP isoenzymes and *Withania somnifera* extracts, as well as the clinical relevance of these findings.

*Eleutherococcus senticosus* is a commonly used adaptogen that is suggested to increase the mental performance of patients with mild fatigue and weakness (A. Panossian and Wikman, 2009b). We identified three cases of adverse bleeding-related events (vaginal hemorrhage, epistaxis, and upper gastrointestinal bleeding) associated with the use of this adaptogen with SSRIs (paroxetine and sertraline) and an SNRI (duloxetine). Possible mechanisms underlying those interactions include the addition of side effects. Studies showed that *Eleutherococcus senticosus* contains dihydroxybenzoic acid, which has antiplatelet activity (Yun-choi et al., 1987; Friedman et al., 2007) and may increase the risk of hemorrhage associated with the use of SSRIs and SNRIs (Zeiss et al., 2021). There is only one study reporting adverse bleeding-related events associated with the use of this adaptogen. Friedman et al. (2007) reported a case of multifocal and recurrent spontaneous subarachnoid hemorrhage caused by the use of *Eleutherococcus senticosus* in combination with other herbal supplements (red clover and dong quai) (Friedman et al., 2007). Cases of adverse bleeding-related events associated with the use of those preparations should be reported in the literature, and herbal medicines should be considered the possible cause of hemorrhage (Friedman et al., 2007). We have also shown a case of the patient presenting increased irritability, agitation, headache, and dizziness when agomelatine was simultaneously used with *Eleutherococcus senticosus*. We hypothesize that this adverse event may be associated with the inhibitory effect of this adaptogen on CYP2C9 and CYP1A2 (Brinker, 2010; Guo et al., 2014), leading to the increased concentration and side effects of agomelatine (Carvalho et al., 2016). Furthermore, irritability and headache have been listed as the side effects associated with the use of *Eleutherococcus senticosus*, indicating the presence of the addition of side effects.

*Tribulus terrestris* is commercialized with indications to improve sexual and athletic performance (Stefanescu et al., 2020). It has been shown that extracts obtained from this plant exhibit inhibitory effects on CYP3A4 (Wang et al., 2022). We have identified three reports of adverse events associated with the concomitant use of this adaptogen and the antidepressants metabolized by this enzyme. Inhibition of CYP3A4 by *Tribulus terrestris* preparations was most likely associated with the increase in the concentration and the severity of side effects of citalopram (generalized pruritus (Citalopram Side Effects, 2023)), escitalopram (galactorrhea (Ravi et al., 2014)), and trazodone (psoriasis relapse (Barth and Baker, 1986)). To the best of our knowledge, there are no studies reporting herb–drug interactions associated with the use of this herb. Our results show that attention should be paid when *Tribulus terrestris* is used with drugs metabolized by CYP3A4.

*Schisandra chinensis* is widely used to treat fatigue and insomnia (Sowndhararajan et al., 2018). The major bioactive substances in these preparations are lignans. This pharmacologically heterogeneous group contains more than 40 particles, of which the most commonly evaluated are: schisandrin, schisandrin A, schisandrin C, deoxyschisandrin, shisanthenol, schisantherin A, gomisins (A, B, C, and N), and wuweizisu C (Sowndhararajan et al., 2018; Seo et al., 2021a). Those substances inhibit numerous cytochrome isoenzymes, including CYP3A4, CYP2B6, CYP2C8, CYP2C9, and CYP2C19, and thus their coadministration with the drugs may result in clinically relevant pharmacokinetic interactions (detailed analysis is presented in Seo et al., 2021a). It has been shown that through the inhibition of CYP3A4, *Schisandra chinensis* extracts increase the concentrations of tacrolimus in liver transplant patients (Jiang et al., 2010) as well as midazolam in rats (Li et al., 2013). In our study, we have found four cases of adverse events related to the use of this adaptogen. We hypothesize that through the inhibition of CYP2B6, CYP2C9, CYP2C9, CYP2C19, and CYP3A4, *Schisandra chinensis* preparations increased the concentration and side effects of bupropion (thrombocytopenia (Altintas et al., 2013) and generalized arthralgia (Ornetti et al., 2004)), amitriptyline (delirium (King and Ashraf, 2018)), and fluoxetine (dysuria (Fluoxetine Side Effects, 2023)).

*Cimcifuga racemosa* is suggested to ameliorate menopausal symptoms such as hot flashes, profuse sweating, anxiety, and insomnia (Mahady, 2005). It has been shown that ethanolic extracts derived from this plant contain eight triterpene glycosides that inhibit CYP3A4, as well as two alkaloids (protopine and allocryptopine) that revealed inhibitory effects on CYP2D6 (J. Li et al., 2011). We have identified three cases of adverse events caused by the concomitant use of this adaptogen and antidepressants metabolized by those cytochromes. We hypothesize that the inhibition of CYP2D6 increased the concentration and side effects of venlafaxine (hyponatremia; additionally associated with the inhibition of CYP3A4), mianserin (restless legs syndrome (Hoque, 2020)), and paroxetine (gynecomastia/mastalgia (Damsa et al., 2003)). In the latter case, the addition of side effects may be involved, as breast pain/enlargement has been reported during *C. racemosa* treatment (Black Cohosh, 2003; Mahady, 2005).

*Coptis chinensis* was traditionally used to treat gastrointestinal symptoms and insomnia (J. Wang et al., 2019). Berberine, one of the most important active constituents of this plant, is being studied for its possible use in the treatment of mood disorders (Fan et al., 2019). Our analysis presented three cases of clinically significant drug–herb interactions associated with the use of *Coptis chinensis*. Their plausible mechanisms involve the inhibition of CYP2D6 and CYP3A4 by berberine (Guo et al., 2012), which increases the concentration and side effects of mirtazapine (edema of lower limbs and myalgia (Lai et al., 2016)), as well as inhibition of CYPD6 (Guo et al., 2012) that increases concentration and side effects of fluoxetine (gynecomastia (Boulenger et al., 2003)) and mianserin (ventricular arrhythmias (Haine et al., 2006)).

*Bacopa monnieri* has been traditionally used as a “brain tonic,” which was intended to enhance memory and concentration (Ramasamy et al., 2014). Studies suggest that extracts derived from this plant can contribute to herb–drug interactions, as they have an inhibitory effect on the activity of many cytochrome isoenzymes such as CYP2C9, CYP2C19, CYP1A2, CYP2D6, and CYP3A4 (Ramasamy et al., 2014). Animal studies have shown that *Bacopa monnieri* extracts increase intestinal absorption and reduce first-pass metabolism of amitriptyline through the inhibition of CYP3A and CYP2C and decrease oral clearance of this drug (Khurshid et al., 2018). To the best of our knowledge, there is only one study presenting adverse events related to the drug interaction with this adaptogen. Acquarulo et al. (2022) showed the case of a 58-year-old patient with Sjogren’s syndrome presenting cholinergic toxicity symptoms (hyperhidrosis, malaise, nausea, and tachycardia) associated with the concomitant use of *Bacopa monnieri* and cevimeline. It has been suggested that the mechanism involved the inhibition of cytochrome isoenzymes responsible for the metabolism of this drug (CYP3A4 and CYP2D6). Clinical improvement has been shown after discontinuation of the supplement (Acquarulo et al., 2022). In this study, we have presented two cases of side effect events associated with the use of this adaptogen and antidepressant treatment, particularly with agomelatine (back pain and hyperhidrosis) and moclobemide (myocardial infarction). *Bacopa monnieri* extracts may increase the concentration and side effects of drugs metabolized by CYP1A2 (agomelatine) and CYP2C19 (moclobemide), thus leading to the aforementioned symptoms. Since *Bacopa monnieri* has an impact on major CYP isoforms responsible for drug metabolism, physicians should be aware of the risk of herb–drug interactions associated with the use of this adaptogen.

*Cordyceps sinensis* is a member of the Ascomycetes fungus family, which grows on the dorsum of caterpillar larvae (*Hepialis armoricanus*). It is commonly used as a dietary supplement with the aim to enhance athletic performance, benefit the immune system, and promote longevity (Hatton et al., 2018). In this study, we have presented the case of upper gastrointestinal bleeding associated with the concomitant use of this adaptogen and sertraline. Plausible mechanisms responsible for this adverse event were the addition of side effects. A recent study has identified two polysaccharides (purified exopolysaccharides and purified intercellular exopolysaccharides) in *Cordyceps sinensis*, which showed dose-dependent inhibition of platelet activation and aggregation (Mao et al., 2022). In addition, it has been clinically observed that the daily intake of *Cordyceps sinensis* may result in prolonged bleeding after surgery (Hatton et al., 2018). Thus, physicians should be aware that the use of this adaptogen may increase the risk of hemorrhage in a group of patients treated with antidepressant drugs revealing antiplatelet effects, such as SSRIs (Laporte et al., 2017).

*Lepidium meyenii* (maca) is popularly referred to as a “natural drug” for the improvement of sexual desire, despite limited evidence to support those claims (Shin et al., 2010). To the best of our knowledge, there are no previous reports of preclinical or clinical drug interactions associated with the use of this adaptogen (Sprouse and Van Breemen, 2016). *In silico* analysis suggests that one of the active compounds of maca (N-(3-methoxybenzyl)-(9Z,12Z,15Z)-octadecatrienamide) may reveal CYP3A4 inhibitory potential (Ibrahim et al., 2022). Through the inhibition of this cytochrome isoenzyme, maca could lead to the increased concentration and side effects of mianserin, leading to the development of restless legs syndrome observed in the reported case in our study. However, a recent *in vitro* study has shown no significant induction or inhibition of maca extracts on CYP3A4 (Y. Zhang et al., 2019). More studies are required to evaluate the risk of herb–drug interactions associated with the use of *Lepidium meyenii* preparations.

*Scutellaria baicalensis* is commonly used in folk medicine to treat depressive symptoms (W. Zhu et al., 2008). Studies indicate that this plant comprises many bioactive compounds, such as baicalein, baicalin, and wogonin, which are associated with pharmacokinetic and pharmacodynamic interactions with a wide range of drugs (Zhou et al., 2021). We have shown the case of a patient who suffered an epileptic seizure as a side effect related to the simultaneous use of sertraline, tramadol, bupropion, and *Scutellaria baicalensis* preparation. Bioactives of this plant present complex interactions with cytochrome isoenzymes responsible for bupropion metabolism. Aqueous extracts of this herb strongly inhibit CYP2C9, while baicalein and luteolin may inhibit CYP2B6 (Noh et al., 2015; Cao et al., 2017; Zhou et al., 2021). As tramadol and bupropion decrease the seizure threshold, the increased concentration of the latter leads to more severe outcomes of the addition of side effects (Davidson, 1989; Boostani and Derakhshan, 2012). However, it is important to emphasize that there is a significant discrepancy between studies evaluating interactions between *Scutellaria baicalensis* active compounds and cytochrome isoenzymes, indicating their contradictory activity (induction or inhibition) (summarized in Zhou et al. (2021)). The composition of the preparation may significantly affect the metabolism of bupropion. For example, a high concentration of baicalin may significantly induce CYP2B6-catalyzed hydroxylation of this drug (Fan et al., 2009).

*Gynostema pentaphyllum* (jiaogulan, herb of immortality) is described as a calming adaptogen providing “longevity and optimum wellbeing.” Gypenosides, one of the most pharmacologically active components of this herb, present significant inhibition of CYP2D6, which is capable of inducing herb–drug interactions. We have identified one side effect event associated with the simultaneous use of *Gynostema pentaphyllum* and an antidepressant metabolized by this cytochrome isoenzyme, which is duloxetine. We hypothesize that through CYP2D6 inhibition, gypenosides increased the concentration and side effects of duloxetine, leading to the occurrence of side effects in the form of lower back pain with increased muscular tension.

Additional mechanisms through which adaptogens interact with other drugs may involve their influence on p-glycoprotein. This protein complex is extensively expressed in the intestinal epithelium and blood–brain barrier where it is responsible for pumping xenobiotics (including drugs) out of the cells (Kim, 2002). Most of the aforementioned herbs contain active compounds which interact with p-glycoprotein. It has been shown that extracts of *Withania somnifera*, *Eleutherococcus senticosus*, *Schisandra chinensis*, *Bacopa monnieri*, *Gynostema pentaphyllum*, and *Scutellaria baicalensis* inhibit the activity of this protein complex. These substances may affect the distribution of antidepressant drugs that are p-glycoprotein substrates. Inhibition of this transport system may lead to an increase in their concentration in the central nervous system and more severe side effects. Antidepressants whose metabolism can be altered by the aforementioned mechanism include sertraline, agomelatine, citalopram, escitalopram, trazodone, and paroxetine (Kim, 2002; O’Brien et al., 2012; Elmeliegy et al., 2020).

The simultaneous use of herbal medicines and prescribed medication is a common phenomenon (Agbabiaka et al., 2018), and the application of adaptogens is becoming popular. Intriguingly, the prevalence of interactions between those preparations and antidepressant drugs was twofold higher than the occurrence of adverse events caused by the interactions of antidepressants with over-the-counter drugs (4%) that were presented in our previous study (Woroń et al., 2022). In clinical practice, the prevalence of those interactions may be much higher. Psychiatrists and physicians may not inquire about the use of adaptogens, as knowledge about the herb–drug interactions in this group of preparations is scarce. Patients may not report the intake of plant-based supplements as they do not consider them as medicines (Agbabiaka et al., 2018). Clinicians should be aware that the risk of the occurrence of herb–drug interactions may be age-related. The mean age of the patients described in the reports was 57 ± 14.3 years, which stays in line with the results of our previous studies (Woroń and Siwek, 2018; Woroń et al., 2019, 2022; Siwek et al., 2020).

There are several limitations to our study. Our study relies on the material of the reported side effects, which may underestimate the frequency of their occurrence since not all physicians provide such reports. Additionally, our analysis covers a relatively narrow time range. The analyzed period was selected on the basis of the availability of the data. The first reports of side effects related to the use of adaptogens were found in January 2021. Since then, the increasing popularity of these preparations has been observed, which translated into an increasing number of adverse events.

## 5 Conclusion and recommendations

- Clinicians should evaluate the presence of overlap between cytochrome P450 isoenzymes involved in the metabolism of adaptogens and antidepressant drugs used by the patients to counteract the occurrence of pharmacokinetic interactions.
- Adaptogen–drug interactions may lead to life-threatening side effects, e.g., upper gastrointestinal bleeding or myocardial infarction as presented in our study.
- Physicians, psychiatrists, and pharmacists should ask patients about the usage of adaptogens and inform them about the risks associated with the concomitant use of those preparations with antidepressants.
- The use of adaptogens should be documented in the patient’s medical records, and the occurrence of herb–drug interactions associated with the use of those preparations should be reported.

## Data Availability

The original contributions presented in the study are included in the article/Supplementary Materials, further inquiries can be directed to the corresponding author.
